# Overexpression of miR-21-5p promotes proliferation and invasion of colon adenocarcinoma cells through targeting *CHL1*

**DOI:** 10.1186/s10020-018-0034-5

**Published:** 2018-07-16

**Authors:** Weihua Yu, Kongxi Zhu, Yulong Wang, Hualong Yu, Jianqiang Guo

**Affiliations:** 1 Department of gastroenterology, the Second Hospital of Shandong University, No.247 Beiyuan Street, Jinan, 250000 Shandong China; 2 Department of Pediatric Internal Medicine, the Second Hospital of Shandong University, Jinan, 250000 Shandong China; 3 Department of Anus and Intestine Surgery, the Second Hospital of Shandong University, Jinan, 250000 Shandong China

**Keywords:** Colon adenocarcinoma, miR-21-5p, *CHL1*, Proliferation, Invasion

## Abstract

**Background:**

This study aims to investigate the effect of miR-21-5p on process of colon adenocarcinoma (COAD) cells and its connection with neural cell adhesion molecule L1 (*CHL1*).

**Methods:**

Different expressions of mRNAs and miRNAs were calculated with microarray analysis. QRT-PCR and western blot were performed to quantify miR-21-5p and *CHL1* expression. Flow Cytometry, MTT assay, colony formation assay, transwell assay and ELISA were performed to evaluate propagation and invasiveness of COAD cells. Dual luciferase reporter assay was employed to scrutinize the relationship between miR-21-5P and *CHL1*. We performed in vivo experiment to detect the impact of miR-21-5p and *CHL1* on COAD tumor growth.

**Results:**

Expression level of miR-21-5p increased in both COAD tissues and cells. MTT and Cell cycle assay showed that overexpression of miR-21-5p accelerated proliferation of COAD cells. Transwell assay indicated that miR-21-5p promoted cell invasion. The result of dual luciferase reporter assay indicated that miR-21-5p targeted *CHL1* directly and inhibited its expression. The result of in vivo experiments showed that down-regulation of miR-21-5p decreased the volume and weight of tumor, while knockdown of *CHLI* stimulated tumor growth.

**Conclusions:**

The overexpression of miR-21-5p can promote propagation and invasiveness of COAD cells through inhibiting the expression of *CHL1*.

## Impact statement

We aimed to investigate the effect of miR-21-5p on process of colon adenocarcinoma (COAD) cells and its connection with *CHL1.*The result showed that the over-expression of miR-21-5p promoted proliferation and invasion of COAD cells through targeting *CHL1.* The findings of our study may assist researchers in discovering the mechanism of progression of COAD and may even help in developing antitumor treatments.

## Background

Colon adenocarcinoma (COAD), a type of human colorectal cancer, is the most common cancers leading to mortality (Phang et al., 2017). Treatments for COAD are classified into four methods, surgery, radiation therapy, chemotherapy and targeted therapy. Optimisation of surgery for patients with localised disease has an important impact on prognosis. Once COAD spreads widely, patients are often given drugs directed towards relieving symptoms to improve quality of life (Cunningham et al., 2010). There are three continually used screening modalities of COAD, including flexible sigmoidoscopy, total colonoscopy and fecal occult blood testing, which have assisted in lowering the mortality caused by COAD. Unexpectedly, cases of COAD in Asian countries have experienced a dramatic uprush during the past decade, while the rate of rectal adenocarcinoma (READ) descends at the speed of 3% every year in several western countries (Kostic et al., 2013). The situation seemed even worse for the latent causes of the origin and the trend of this regional malignancy have not been completely elaborated yet.

To control COAD, therapeutic and worthy-of-promotion targets should be causally related to the disease and obey the designed interventions of the corresponding therapy. Besides, desired biomarkers should be easy to measure, detect and related to clinical results. MicroRNAs (miRNAs) could meet both standards described above. MiRNAs are small RNAs with the length of 21 to 25 nucleotides, which often take part in the regulation of progression of cell cycle, differentiation, apoptosis as well as tumorigenesis (Bartel, 2004). MiRNA levels are altered in most types of tumor (Volinia et al., 2006). It has been shown that experimental operations of specific miRNAs regulate development of tumor in mouse-model systems (Georgantas 3rd et al., 2007; Wang et al., 2006; Dews et al., 2006). Previous study revealed that overexpression of miR-141 resulted in proliferation of COAD cells (Ding et al., 2017). It’s also reported that miR-1297 inhibited COAD cell migration, invasion and proliferation in vivo (Chen et al., 2014). If abnormal miRNA expression is a causal factor to carcinogenesis, regulating miRNA expression may be a new strategy in cancer treatments.

MicroRNA-21 (miR-21), located in 17q23.1, is an oncogenic microRNA that regulates some cancer-related gene expressions such as *TPM1, PTEN*, *PDCD4*, *TIMP3*, *RHOB* and *PDCD* and has been confirmed to be highly expressed in COAD (Bovell et al., 2013; Leone et al., 2013; Martin del Campo et al., 2015; Xu et al., 2014). MiR-21 also plays a role in several signaling pathways such as, Wnt/β-catenin, PTEN/PI3K/AKT andRAS/MEK/ERK (Xiong et al., 2013; Lan et al., 2015). Several studies confirmed that increasing expression of miR-21 was related to the prognosis of COAD in stage I (Kjaer-Frifeldt et al., 2012; Oue et al., 2014). Most researches uncovered an elevated expression of miR-21 in colorectal cancerous tissues in comparison to paracancerous tissues (Oue et al., 2014; Tokarz & Blasiak, 2012; Nielsen et al., 2011; Chen et al., 2013), while there are few researches on a specific type of cancer cells in colorectal cancer and the mechanism of how miR-21 influences progression of COAD still remains unclear. Therefore, it is essential to carry out deeper analyses of miR-21 and COAD.

Neural cell adhesion molecule L1 (*CHL1*) is a neural adhesion molecule, which may function in signal transduction pathways and also participate in normal tissues and various of human cancers (He et al., 2013). It was reported that *CHL1* had a close relationship with mental retardation in 3p-syndrome (Wei et al., 1998), which was associated with schizophrenia in Chinese population (Chen et al., 2005). Alteration of *CHL1* has been implicated in the development of different cancers, including colon cancer (Gavert et al., 2005). *CHL1* is verified to inhibit invasive growth and able to suppress further metastatic spread in COAD (Senchenko et al., 2011). Moreover, some researches verified that miR-21 targeting *CHL1* to inhibit the neuroblastoma cell growth (Li et al., 2016a). However, few studies have been carried out to research the molecular mechanism between *CHL1* and miR-21in COAD at present.

Our study explored dysregulation of miR-21-5p in both COAD tissues and cells. MTT assay, flow cytometry assay and transwell assay were employed to show the effect of miR-21-5p on proliferation, cell cycle and invasion. Furthermore, we investigated the relationship between miR-21-5p and *CHL1* through dual-luciferase report and western blot. Moreover, we detected the impact of miR-21-5p/*CHL1* axis on proliferation by in vivo experiment. The findings of our study may assist researchers in discovering the mechanism of progression of COAD and may even help in developing antitumor treatments.

## Methods

### Samples collection

43 paired of cancerous and adjacent tissues were provided by COAD patients. Normal colonic epithelial cells and COAD cell lines CW-2, T84, SW1116, LoVo were offered by BeNa Culture Collection (BNCC, Beijing, China). The Second Hospital of Shandong University approved the study and all informed consent had been signed and gathered beforehand.

### Cell culture

Cells were cultured in Dulbecco’s Modified Eagle Medium (DMEM) together with additional 100 U/ml penicillin/streptomycin and 10% Fetal Bovine Serum (FBS, Invitrogen, Gaithersburg, MD, USA). The cells were incubated in a humidified incubator with 5% CO_2_ at 37 °C.

### Microarray analysis

The microarray data of differentially expressed miRNAs and mRNAs in COAD tissues was analyzed from the Cancer Genome Atlas (TCGA) database (https://cancergenome.nih.gov/). Differentially expressed miRNAs and mRNAs were screened with a t-test (*P* < 0.05) combined with fold change (FC) (log_2_ (FC) > 2 for up-regulated, and log_2_ (FC) < − 2 for down-regulated).The target genes and the binding sites of miRNAs were predicted by TargetScan 7.1 database (www.targetscan.org).

### Quantitative real time polymerase chain reaction (qRT-PCR)

Total RNA was extracted by Trizol Reagent (Invitrogen). RNAs were reversely transcribed into cDNA using Takara Reverse Transcription System (Takara, Dalian, China) before qPCR was carried out. The reaction program was set initially at 95 °C for 30 s, followed by other 40 cycles composed of 95 °C for 10 s and 60 °C for 30 s, with a final dissociation stage of 95 °C for 15 s, 60 °C for 1 min and 95 °C for 15 s. GAPDH and U6 acted as internal control respectively for *CHL1* and miR-21 measurement. The quantitative results were yielded by the relative quantification approach (${2}^{-\Delta  \Delta  {C}_t}$). Primer sequences were designed by Sangon Biotech (Shanghai, China) and listed in Table 1.

**Table 1:** Primer Sequences

Gene	Primer	Sequences
CHL1	Forward	5’-CGGACTAGTCTATACATCCACAGGGTT-3’
	Reverse	5’-CCCAAGCTTTCTTTAGCCACTTCAGTT-3’
miR-21-5p	Forward	5’-ACACTCCAGCTGGGTAGCTTATCAGACTGA-3’
	Reverse	5’-TGGTGCGTGGAGTCG-3’
U6	Forward	5’-CTCGCTTCGGCAGCACA-3’
	Reverse	5’-AACGCTTCACGAATTTGCGT-3’
GAPDH	Forward	5’-GGGTGTGAACCATGAGAAGT-3’
	Reverse	5’-GGCATGGACTGTGGTCATGA-3’

### Cell transfection

MiR-21-5p mimics, mimics negative control (mimics NC), miR-21-5p inhibitor, inhibitor negative control (inhibitor NC), *CHL1* siRNA, pcDNA3.1 *CHL1*, siRNA negative control(siRNA NC)and miR-21-5p inhibitor + *CHL1* siRNA were manufactured by Sangon Biotech. Cells were plated and cultured in complete medium at 37 °C for 24 h. Next, cells were transferred into serum-free medium, mixed with plasmid and liposome transfection reagent. Cells were prepared by rinsing with serum-free medium. The transfection was conducted by Lipidosome™2000 (Invitrogen). Transfection efficiency was detected after 48 h.

### Western blot

Total protein in cells was extracted by radio immunoprecipitation buffer (RIPA, Sigma-Aldrich, St. Louis, MO, USA) and quantified by bicinchoninic acid (BCA, Pierce, Rock ford, IL, USA). After loading the equivalent amount of protein to SDS-PAGE (Bio-Rad, Hercules, CA, USA), proteins were then transferred to polyvinylidene fluoride (PVDF, Invitrogen) membranes. Followed blocked by 5% skim milk for 1 h, primary rabbit monoclonal antibody *CHL1* solution (Epitomics, Burlingame, CA, USA) was placed into the membranes. After 2 h incubation, the membranes were incubated with second antibody: horseradish peroxidase (HRP) conjugated goat anti-rabbit (1:1000, Proteintech Group, Rosemont, IL, USA) at room temperature for 1 h. After washed by Tris Buffered Saline Tween (TBST), proteins were observed with an enhanced chemiluminescent (ECL, Thermo Scientific, Waltham, MA, USA) followed by exposure to X-ray films. The murine monoclonal antibody actin (1:2000, Proteintech Group) was used as internal reference.

### MTT assay

2 × 10^3^ cells were sowed into 96-well plates (100 μL/well) with 20 μL/well of MTT (5 mg/mL, Sigma-Aldrich) and incubated for 4 h. After washed by PBS, cells were dissolved and crystallized by 150 μL dimethyl sulfoxide (DMSO, Sigma-Aldrich). The optical density (OD) was detected by a microplate reader (Molecular Devices, Sunnyvale, CA, USA) at wavelength of 570 nm.

### Flow cytometry assay

Cells were re-suspended in 100 μL PBS and fixed with 400 μL anhydrous ethanol for 1 h. After incubated at 37 °C for 1 h, 500 μL PBS was added and ethanol was removed. After incubated with 100 μg/ml RNase A (Sigma) in the dark at 37 °C for 30 min, 5 μL of Annexin V-fluorescein isothiocyanate (Annexin V-FITC) and propidium iodide (PI) (Becton Dickinson, NJ, USA) were added into the suspension for 15 min at room temperature in the dark. Each tube was supplemented with 400 μL binding buffer and cells were calculated by a flow cytometry (Beckman FC 500 MCL/MPL, Beckman Coulter, Miami, FL, USA).

### Transwell assay

The upper surface of transwell chambers were covered with 40 μL Matrigel (BD Biosciences, San Jose, CA, USA) diluted with DMEM in proportion 1:3 for 24 h at 37 °C. After lysed with trypsin, cells were centrifuged, collected and re-suspended in 200 μL serum-free medium. Then the upper chambers were added with the cell suspension and 600 μL medium containing 20% FBS (Invitrogen) was added onto the substratum of chambers. After 24 h cultivation, cells were then fixed by methyl alcohol for 20 min. In the end, we used 0.1% crystal violet to stain the cells, and counted the cells under a microscope (Nikon, Japan).

### Colony formation assay

Cells were collected when they were in logarithmic phase. After complete medium was removed, cells were washed by PBS before stained with 0.1% crystal violet for 30 min. Methanol was utilized to fix the cells. The colony cells were counted under an optical microscope (Nikon).

### Enzyme-linked immunosorbent assay (ELISA)

Human MMP-2 Quantikine ELISA kit (R&D Systems, USA) and human MMP-9 ELISA kit (Abnova, Taiwan) were applied to test the expression of MMP-2 and MMP-9 respectively. After transfection, cells were incubated with fresh DMEM for 24 h, after which the culture medium was collected and centrifuged at 2000 rpm. Clarified medium was analyzed with ELISA kit (Institut Pourquier).

### Dual luciferase reporter assay

*CHL1* wt and mut were both synthesized by Sangon Biotech, which were transfected in LoVo cells. After post-transfection for 48 h, luciferase activities were analyzed by dual-luciferase reporter assay system (Promega, Madison, WI, USA) as the protocol suggested. The ratio of firefly to renilla luciferase signal was used to normalize efficiency of firefly activity in intra experimental transfection.

### Mouse xenograft model

We divided the 4-weeks-old BALB/C athymic female nude mice into 5 groups (4 mice each group), and injected them with cells transfected by miR-21-5p inhibitor, miR-21-5p inhibitor negative control, *CHL1* siRNA, siRNA negative control, miR-21-5p inhibitor together with *CHL1* siRNA respectively. 1 × 10^7^ treated LoVo cells were subcutaneously injected into the back of each subject. Then tumor volume measurement of nude mice in all groups was performed with calipers every week using the following method: volume = (length × width^2^) /2, with each parameter standing for the maximum and the minimum diameter respectively. The weight of tumor was measured after 4 weeks.

### Statistical analysis

Statistical results were assessed by SPSS18.0 and all data were displayed in the form as mean ± standard deviation. Student t-test was employed to evaluate the difference between two groups, while one-way ANOVA analysis was used to compare multiple groups. *P* < 0.05 indicated statistically significance.

## Results

### Clinicopathological features of COAD patients

43 samples of COAD patients were used in this study. There were 32 samples with high expression of miR-21-5p and 11 samples with low expression of miR-21-5p. As shown in Table 2, miR-21-5p had close relationship with clinical stage, lymph node metastasis and distant metastasis of the patient samples (all *P* < 0.05). However, it seems that there was no significant correlation between the expression of miR-21-5p and gender or age (all *P* > 0.05).

**Table 2:** Clinicopathological features of COAD patients

Characteristics		The expression of miR-21-5p		
		high expression(*n* = 32)	low expression(*n* = 11)	*P* value
Gender	Male	18	8	0.335
	Female	14	3	
Age(years)	< 55	15	6	0.661
	≥55	17	5	
Clinical stage	I-II	11	8	0.027*
	III-IV	21	3	
Lymph node metastasis	Absence	20	3	0.043*
	Presence	12	8	
Distant metastasis	Absence	19	2	0.018*

Data were compared by Chi-square test, *, *P* < 0.05

### MiR-21-5p highly expressed in COAD tissues and cells

**Fig. 1:**
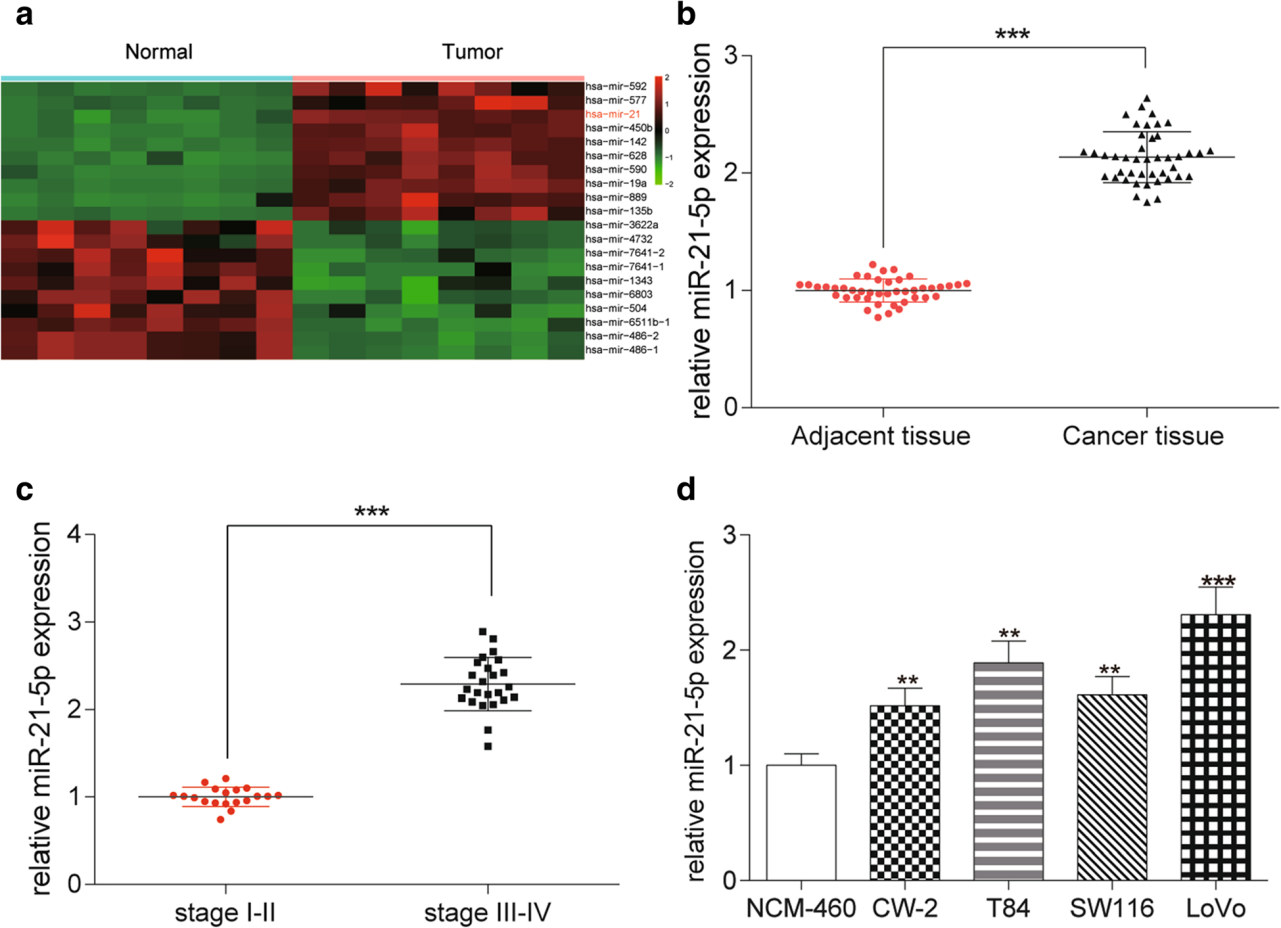
MiR-21-5p highly expressed in COAD tissues and cells. **a** The heat map showed that miR-21-5p was high expressed in COAD tissues compared with normal tissues. **b** The relative expression of miR-21-5p in cancer tissues was much higher than that in adjacent tissues detected by qRT-PCR. ^***^P < 0.001, compared with adjacent tissue. **c** The expression of miR-21-5p in stage III-IV was notably higher than that in stage I-II detected by qRT-PCR. ^***^P < 0.001, compared with stage I-II. **d** The expression of miR-21-5p in COAD cell lines (CW-2, T84, SW116, LoVo) was higher than that in normal colonic epithelial cell line (NCM-460) detected by qRT-PCR, which reached the highest level in LoVo cell line. ^**^P < 0.01, ^***^P < 0.001, compared with NCM-460

Microarray analysis was used to identify differentially expressed miRNAs between COAD tissues and normal tissues. The results showed that miR-21-5p expression in the COAD samples was up-regulated by about 6.22-fold compared to the normal adjacent tissues (*P* < 0.001, Fig. 1a). The result of qRT-PCR indicated that the expression level of miR-21-5p in colorectal cancer tissues was higher than that in adjacent normal tissues, which was more active in the higher pathological grade (both *P* < 0.001, Fig. 1b-c). The expression level of miR-21-5p was also remarkably up-regulated in 4 kinds of COAD cells, especially in LoVo cells compared with normal colonic epithelium cells NCM-460 (*P* < 0.001, Fig. 1d). Thus, LoVo cell line was chosen to be employed in the subsequent experiments.

### MiR-21-5p promoted the propagation and invasiveness of COAD cells

**Fig. 2:**
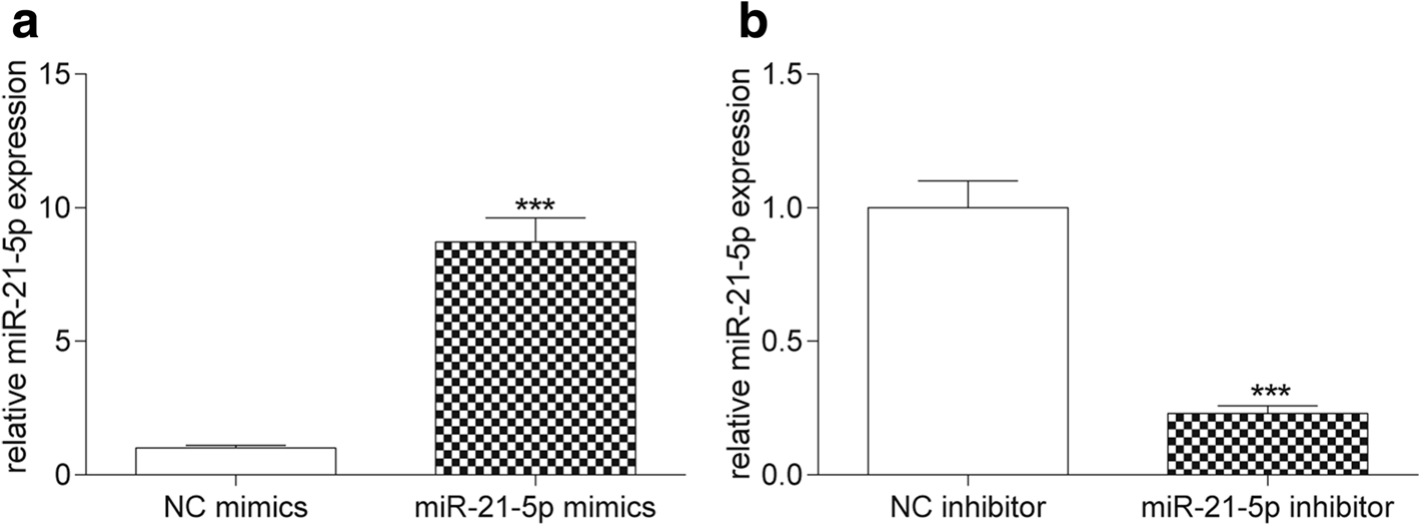
The transfection of miR-21-5p mimics and miR-21-5p inhibitor. **a** The result of qRT-PCR showed that miR-21-5p expression in miR-21-5p mimics was much higher than that in NC mimics. ^***^P < 0.001, compared with NC mimics. **b** MiR-21-5p expression in miR-21-5p inhibitor was significantly lower than that in NC inhibitor. ^***^P < 0.001, compared with NC inhibitor

**Fig. 3:**
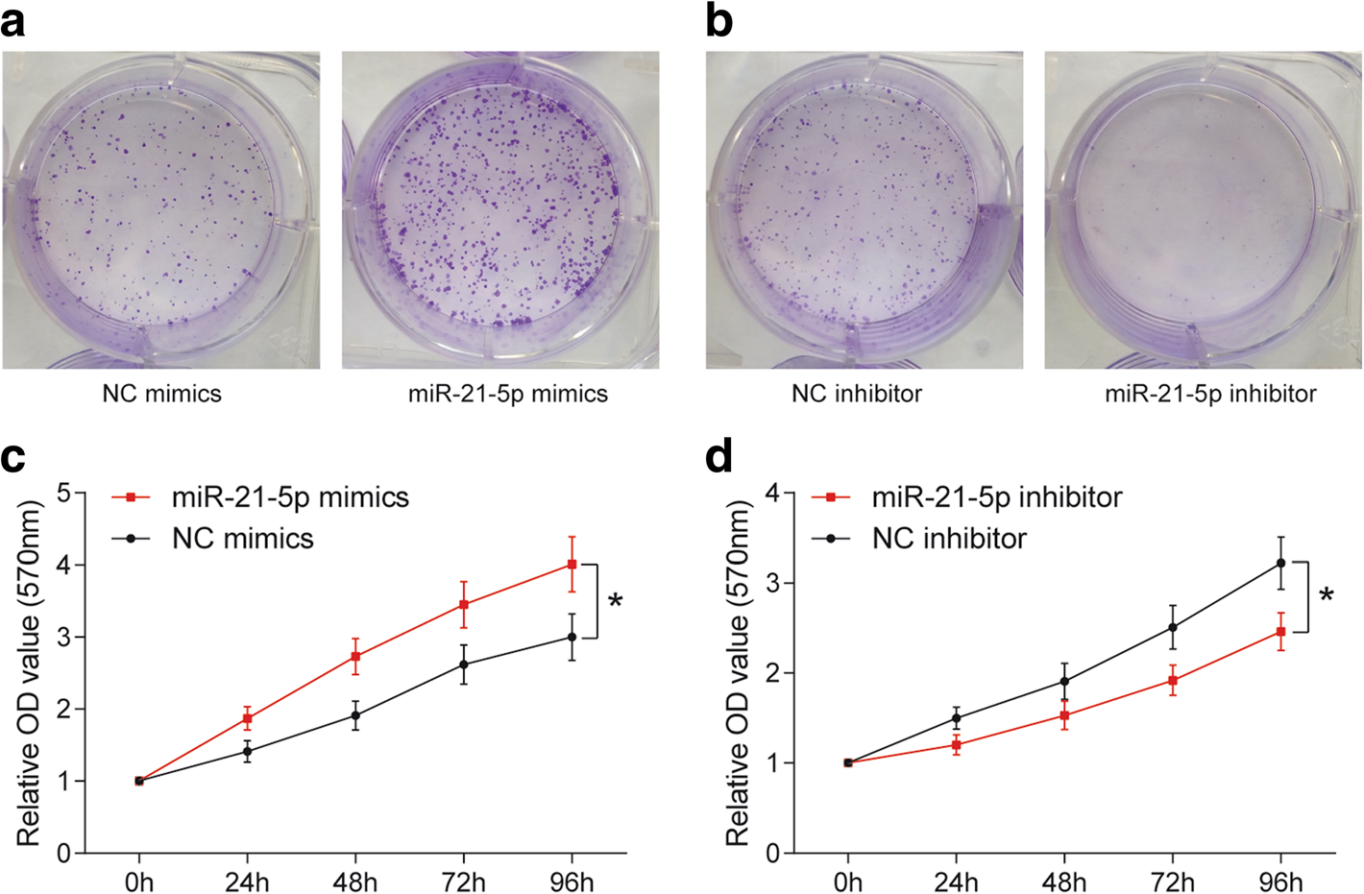
MiR-21-5p promoted COAD cell proliferation. **a**, **b** According to the results of colony formation assay and MTT assay, colony cells in miR-21-5p mimics group were more than that in NC mimics group since 48 h transfection. ^*^P < 0.05, compared with NC mimics. **c**, **d** Colony cells in miR-21-5p inhibitor group were less than that in NC inhibitor group. ^*^P < 0.05, compared with NC inhibitor group

**Fig. 4:**
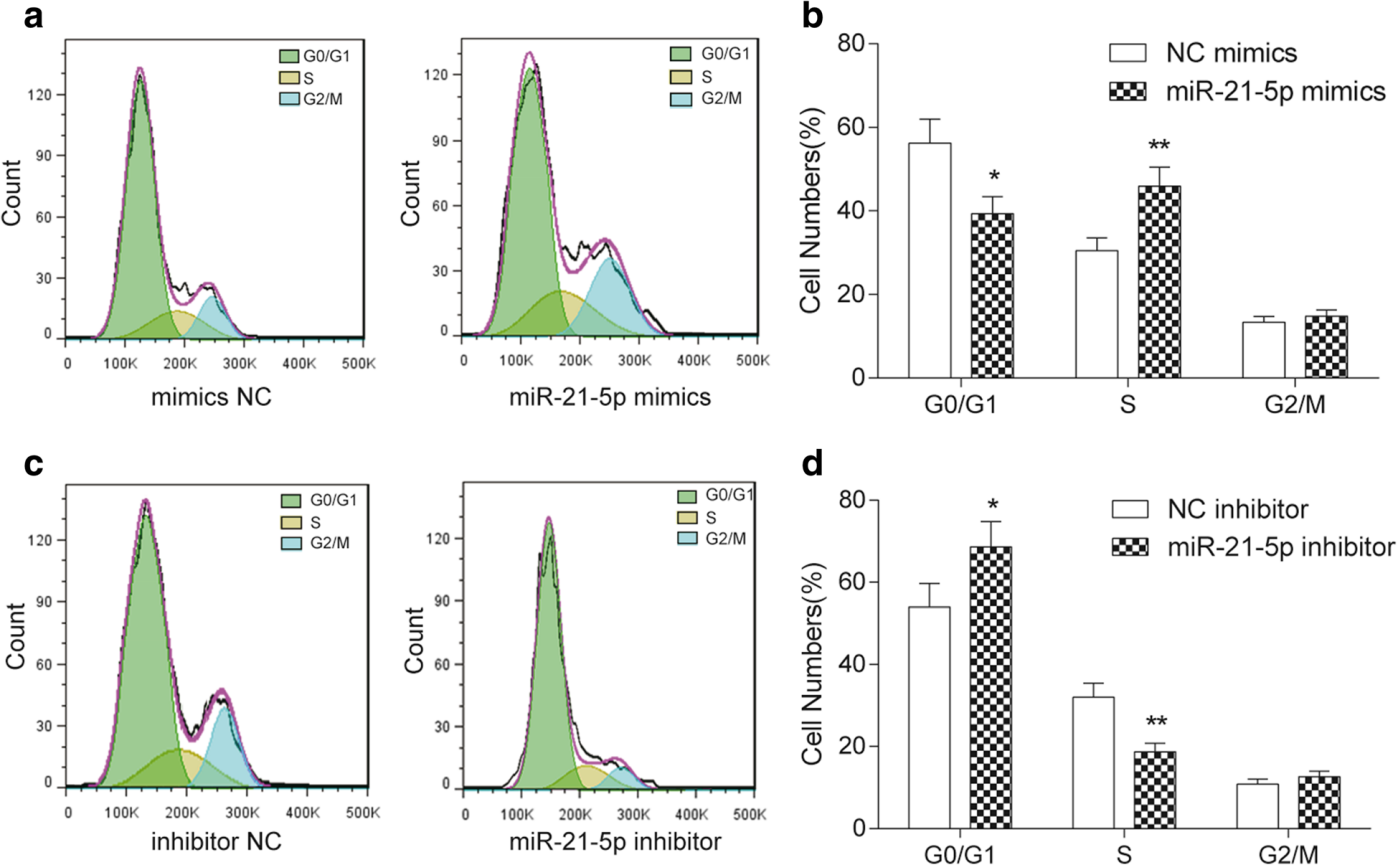
MiR-21-5p promoted the cell cycle of COAD cells. **a**, **b** Compared with NC mimics, more cells in miR-21-5p mimics group were arrested in S phase and less cells appeared in G0/G1 phase. ^*^P < 0.05, ^**^P < 0.01, compared with NC mimics group. **c**, **d** Compared with NC inhibitor, more cells appeared in G0/G1 phase and less cells were arrested in S phase. ^*^P < 0.05, ^**^P < 0.01, compared with NC inhibitor group

**Fig. 5:**
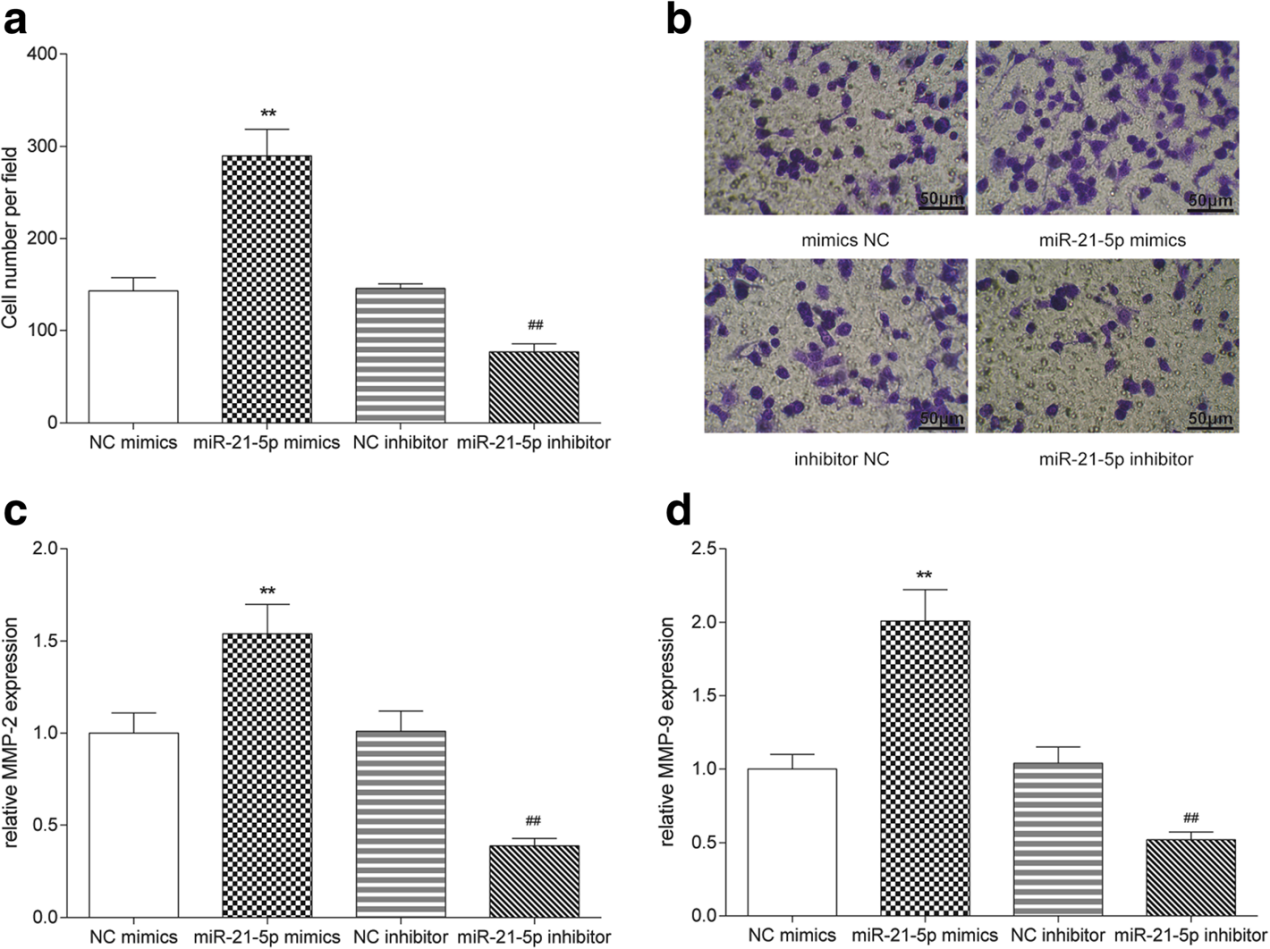
MiR-21-5p promoted the invasion of COAD cells. **a**, **b** Cell number in miR-21-5p mimics group was more than that in NC mimics group, while that in miR-21-5p inhibitor group was less than that in NC inhibitor group detected by transwell assay. The scale bar was 50 μm. **c**, **d** The expressions of invasiveness-related factor MMP-2 and MMP-9 in miR-21-5p mimics group were higher than that in NC mimics group, while those in miR-21-5p inhibitor group were lower than that in NC inhibitor group detected by ELISA. ^**^P < 0.01, compared with NC mimics group, ^##^*P* < 0.01, compared with NC inhibitor group

MiR-21-5p expression was up-regulated followed transfected with miR-21-5p mimics (*P* < 0.001, Fig. 2a), while it was down-regulated after transfected with miR-21-5p inhibitor (*P* < 0.001, Fig. 2b). According to the results of colony formation and MTT assay, with the passage of time, overexpression of miR-21-5p facilitated the propagation of COAD cells (*P* < 0.05, Fig. 3a & c), while inhibition of miR-21-5p suppressed COAD cells proliferation (*P* < 0.05, Fig. 3b & d). Flow cytometry result showed that miR-21-5p mimics promoted the process of CODA cell cycle in S phase (*P* < 0.01), while miR-21-5p inhibitor restrained the process of COAD cell cycle at G0/G1 phase (*P* < 0.05, Fig. 4). The results illustrated that overexpression of miR-21-5p can accelerate proliferation of COAD cells. Transwell assay indicated that overexpression of miR-21-5p promoted the invasion of COAD cells (*P* < 0.01, Fig. 5a), while inhibition of miR-21-5p suppressed it (*P* < 0.01, Fig. 5b). The result of ELISA verified that miR-21-5p stimulated the expression of invasiveness-related factor MMP-2 (*P* < 0.01, Fig. 5c) as well as MMP-9 (*P* < 0.01, Fig. 5d), which were suppressed by miR-21-5p inhibitor. Therefore, miR-21-5p can promote the proliferation and invasion of COAD cells.

### MiR-21-5p inhibited the expression of *CHL1*

**Fig. 6:**
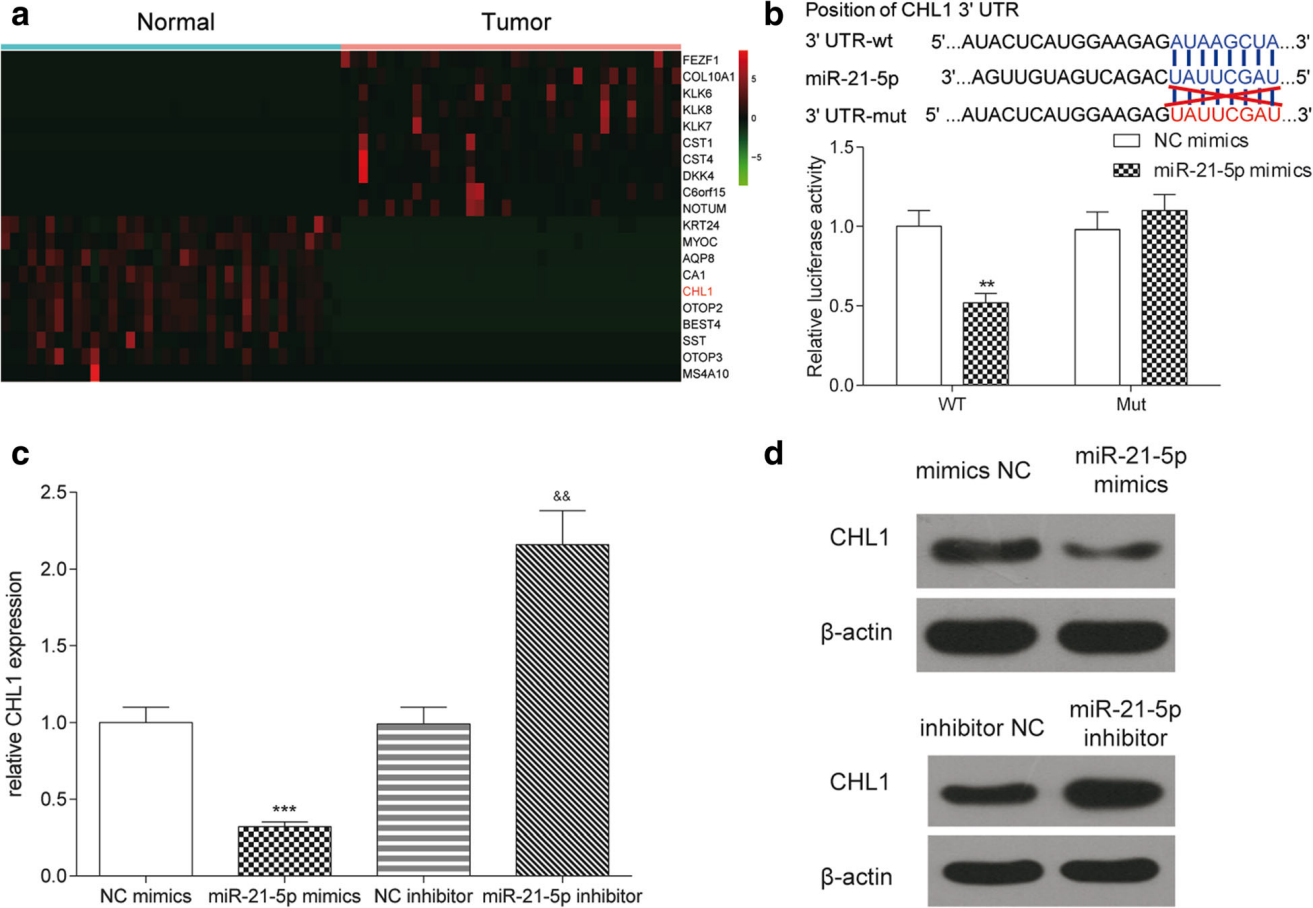
MiR-21-5p inhibited the expression of *CHL1*. **a** The heat map showed that the expression of *CHL1* in COAD tissues was lower than that in normal tissues. **b** TargetScan predicted that there was a binding site for miR-21-5p in the 3’UTR of *CHL1*. The result of dual-luciferase reporter assay showed that the luciferase activity in miR-21-5p mimics and *CHL1* 3’UTR-wt co-combination group was lower than that in NC mimics and *CHL1* 3’UTR-wt co-combination group. In the meantime, there was no significant difference between miR-21-5p mimics and *CHL1* 3’UTR-mut co-combination group NC mimics and *CHL1* 3’UTR-mut co-combination group. **c**, **d** *CHL1* expression in miR-21-5p mimics group was much lower than that in NC mimics group, while that in miR-21-5p inhibitor group was higher than that in NC inhibitor group. ^**^P < 0.01, ^***^P < 0.001, compared with NC mimics group, ^&&^*P* < 0.01, compared with NC inhibitor group

TargetScan was used to predict the target mRNA of miR-21-5p and the outcome showed that a binding site of miR-21-5p existed in the 3’UTR region of *CHL1* (Fig. 6b). We screened the top 10 differentially expressed mRNAs by microarray analysis. The results of microarray analysis indicated that the expression of *CHL1* was down-regulated by 2.57-fold compared with normal tissues (*P* < 0.001, Fig. 6a). Since *CHL1* has been reported as a tumor suppressor (Martin-Sanchez et al., 2017), we supposed that miR-21-5p may promote tumor development via inhibiting *CHL1* expression. Dual luciferase reporter assay was conducted to reconfirm the predicted targeting relationship between miR-21-5p and *CHL1* (*P* < 0.01, Fig. 6b). Overexpression of miR-21-5p significantly inhibited *CHL1* expression (*P* < 0.001), while inhibition of miR-21-5p up-regulated *CHL1* expression (*P* < 0.01, Fig. 6c-d). Hence, miR-21-5p inhibited *CHL1* expression by binding to 3’UTR of *CHL1*.

### *CHL1* inhibited progression of COAD cells

**Fig. 7:**
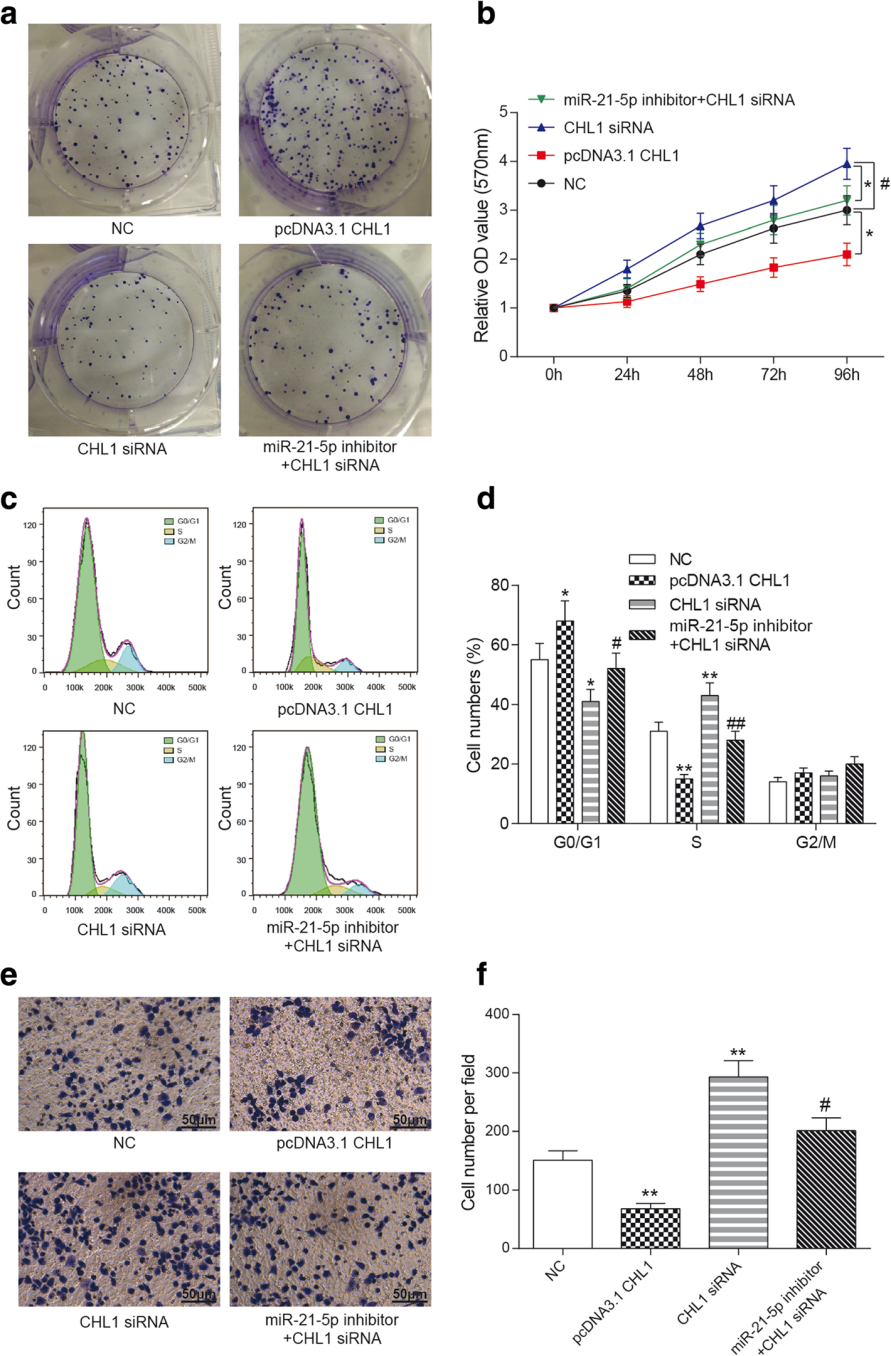
*CHL1* inhibited COAD cells development. **a** Colony formation assay indicated that *CHL1* suppressed COAD cells growth. **b** MTT assay revealed that overexpression of *CHL1* inhibited cell viability, while knockdown of *CHL1* enhanced the proliferation ability of COAD cells. **c**, **d** The result of flow cytometry suggested that *CHL1* arrested cell cycle in G0/G1 phase, while deletion of *CHL1* increased cells in S phase. **e**, **f** Transwell assay was performed to detect invasion ability. The cell number in pcDNA3.1 *CHL1* group was increased, while that in *CHL1* siRNA group was decreased compared with control group. The scale bar was 50 μm. ^*^P < 0.05, ^**^P < 0.01, compared with NC group. ^#^*P* < 0.05, ^##^*P* < 0.01, compared with *CHL1* siRNA group

After transfection, colony formation and MTT assay was used to evaluate cell propagation. The results indicated that cell viability was inhibited by pcDNA3.1 *CHL1* (*P* < 0.05), while it was enhanced by deletion of *CHL1* (*P* < 0.05). In addition, the effect of *CHL1* siRNA was rescued by miR-21-5p inhibitor (*P* < 0.05, Fig. 7a-b). The result of flow cytometry assay revealed that the cells were arrested in G0/G1 phase in pcDNA3.1 *CHL1* group (*P* < 0.05), while the number of cells in S phase increased in *CHL1* siRNA group compared with NC group (*P* < 0.01) and the cells in S phase decreased after co-transfected with *CHL1* siRNA and miR-21-5p inhibitor (*P* < 0.05, Fig. 7c-d). Transwell assay proved that the ability of invasion of COAD cells was attenuated in pcDNA3.1 *CHL1* group (*P* < 0.01). However, in *CHL1* siRNA group, cell invasion ability was enhanced (*P* < 0.01), and it was suppressed by miR-21-5p inhibitor (*P* < 0.05, Fig. 7e-f). As a result, *CHL1* suppressed COAD growth as well as invasion, and blocked cell cycle at G0/G1 phase.

### MiR-21-5p regulated COAD tumor growth in vivo through inhibiting *CHL1* expression

**Fig. 8:**
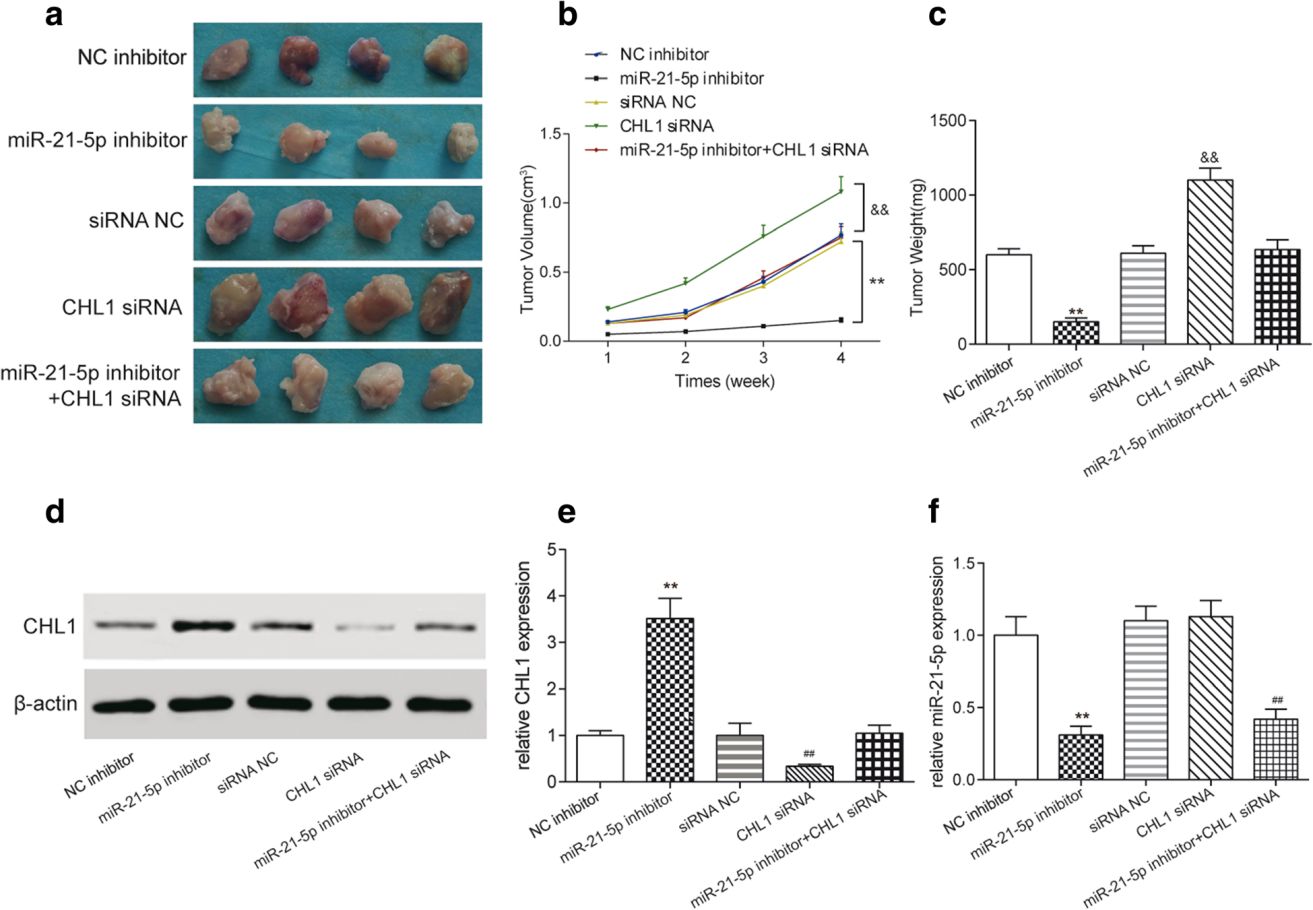
MiR-21-5p promoted COAD tumor growth in vivo through inhibiting *CHL1* expression. **a**-**c** The volume and weight of tumors in miR-21-5p inhibitor group were much lower than that in NC inhibitor group, while that in *CHL1* siRNA group was higher than that in siRNA NC group. ^**^P < 0.01, compared with NC inhibitor group, ^&&^*P* < 0.01, compared with siRNA NC group. **d**, **e** *CHL1* expression in miR-21-5p inhibitor group was much higher than that in NC inhibitor group, while that in *CHL1* siRNA group was lower than that in siRNA group. Meanwhile, there was no significant difference between NC inhibitor group, siRNA NC group and miR-21-5p inhibitor+*CHL1* siRNA co-combination group. ^**^P < 0.01, compared with NC inhibitor group, ^##^*P* < 0.01, compared with siRNA NC group. **f** The expression of miR-21-5p was down-regulated by miR-21-5p inhibitor in vivo evaluated by qRT-PCR. ^**^P < 0.01, compared with NC inhibitor group, ^##^*P* < 0.01, compared with siRNA NC group

The result of in vivo experiment indicated that inhibition of miR-21-5p suppressed COAD tumor growth and knockdown of *CHL1* promoted the growth of COAD tumors (Fig. 8a-c). The expression of miR-21-5p and *CHL1* in tumor was accessed by western blot and qRT-PCR. The result of western blot showed that the expression of *CHL1* was up-regulated by inhibiting miR-21-5p (*P* < 0.01, Fig. 8d-e). The expression of miR-21-5p was also down-regulated by miR-21-5p inhibitor in vivo (*P* < 0.01, Fig. 8f).Therefore, miR-21-5p promoted the tumor growth by inhibiting *CHL1* expression in vivo as well.

## Discussion

In this research, we explored the correlation between miR-21-5p and clinicopathological features of COAD patients as well as miR-21-5p expression in COAD cells. In our experiments, the results illustrated that miR-21-5p stimulated the propagation and invasiveness of COAD cells. Moreover, we detected that miR-21-5p inhibited *CHL1* expression. In the meantime, we explored in vivo experiment which confirmed that miR-21-5p promoted the tumor growth by inhibiting *CHL1* expression.

COAD is a common type of colorectal cancer, and it is the fourth most common cause of cancer-related death around the world. Although chemotherapy is generally effective in weakening growth of tumor cells and impeding metastasis, it often loses virtue in advanced stages of COAD due to development of chemoresistance (Germani et al., 2014), which leads to recurrence of cancer or even death of patients. MiRNAs are often dysregulation in various types of tumor, which play crucial roles in tumorigenesis (Lambert & Becker, 1989; Hur, 2015). Our findings suggested that miR-21-5p highly expressed in COAD cells. Consistent with our results, a previous study indicates that miR-21 expression increased in COAD tissues as well as COAD cell lines (Liu et al., 2013). A recent study finds that miRNA-21 promotes invasion, migration and proliferation of COAD (Li et al., 2016b), which indicates that miR-21 plays a vital role in COAD development. To further explore the impact of miR-21-5p on cell activity of COAD, we did MTT assay and transwell assay, which showed that miR-21-5p promoted the propagation and invasiveness of COAD cells. These results corresponded with the previous reports reveal that miR-21 induces invasion and proliferation of COAD cells (Asangani et al., 2008). Moreover, we found that miR-21-5p over-expression enhanced the development of tumor in vivo*,* which coincided with a previous report that over-expression of miR-21 in COAD cells fortified their tumorigenic potential (Xu et al., 2014).

*CHL1*, highly related to the L1 protein, is an adhesion molecule of cells. A great number of studies have shown that miRNAs were able to target several transcription factors to regulate the metabolism and progression of cells (Li et al., 2017; Cao et al., 2017; Zhuo et al., 2017; Qiu & Dou, 2017). In order to ascertain the regulatory relationship between miR-21-5p and *CHL1*, we employed TargetScan as well as dual luciferase reporter assay and found that *CHL1* 3’UTR has a binding site with miR-21-5p. Moreover, the expression level of *CHL1* was up-regulated by inhibiting miR-21-5p. Previous study verified that *CHL1* gene was one of the putative tumor suppressor genes localized on human chromosome 3 (Qin et al., 2008). In our experiments, knockdown of *CHL1* promoted the tumor growth. Previous report also revealed that miR-21 promotes the propagation and invasiveness of neuroblastoma cells by suppressing *CHL1* (Li et al., 2016a).

Even though we have observed some meaningful results in our study, there were also some clear limitations. For example, our study only looked into the functions of miR-21-5p in promoting proliferation and invasion of COAD cells, whereas the underlying detailed mechanism of miR-21-5p in COAD cells remained blurry which was quite essential to completely understand the pathogenesis of COAD. In addition, miR-21-5p was controlled by one gene called miR-21 and in previous studies, miR-21 has been proved to be capable of targeting many tumor suppressors such as *PTEN* (Meng et al., 2007), *RECK* (Gabriely et al., 2008), *BTG2* (Liu et al., 2009) and *TP63* (Papagiannakopoulos et al., 2008). The correlation of miR-21 and its other target genes in COAD cells need to be further studied.

## Conclusions

In conclusion, our experiments demonstrated that over-expression of miR-21-5p enhanced proliferation and invasion of COAD cells through targeting *CHL1*. Nevertheless, further research is necessarily required to deeply and comprehensively understand the mechanism of miR-21 on COAD.

## Abbreviations

Annexin V-FITC: Annexin V-fluorescein isothiocyanate
BCA: Bicinchoninic acid
BNCC: BeNa Culture Collection
*CHL1*: Cell adhesion molecule L1
COAD: Colon adenocarcinoma
DMEM: Dulbecco’s Modified Eagle Medium
DMSO: Dimethyl sulfoxide
ECL: Enhanced chemiluminescent
FBS: Fetal Bovine Serum
FC: Fold change
inhibitor NC: Inhibitor negative control
mimics NC: mimics negative control
miR-21: MicroRNA-21
miRNAs: MicroRNAs
OD: Optical density
PI: Propidium iodide
PVDF: Polyvinylidene fluoride
READ: Rectal adenocarcinoma
RIPA: Radio immunoprecipitation buffer
RP: Horseradish peroxidase
siRNA NC: siRNA negative control
TBST: Tris Buffered Saline Tween
TCGA: Cancer Genome Atlas
